# Restoration of Traditional Children’s Play in Iranian Nomadic Societies (Case Study of Kohgilouyeh and Boyer Ahmad)

**DOI:** 10.3390/children2020211

**Published:** 2015-05-29

**Authors:** Laleh Taheri, Golshan Chahian

**Affiliations:** 1 Architect, from the nomadic tribe of Boyer Ahmad, CENESTA, Shiraz, Iran; 2 Ecology and sustainable landscape planning and designer, CENESTA, Shiraz, Iran; E-Mail: golshan.chahian@gmail.com

**Keywords:** Kohgilouyeh and Boyer Ahmad, Iran, customary culture, nomadic children, indigenous play, modern play, social change, emotional wellbeing, physical health, mental health

## Abstract

This article aims to provide an insight into play as an important aspect of children’s lives in an under-studied area of Iran. Our observations focus on the province of Kohgilouyeh and Boyer Ahmad with its ancient nomadic cultures. Through first-hand knowledge and lived experiences, supplemented by available literature, we seek to look at children’s games in the frame of culture change, exploring their relationship with children’s health and wellbeing. Play, as in every region in the world, conveys and reflects the dominant culture and teaches the values of the society in which the children live in the here and now and in which they will have to function as adults. Yet, types of play are not static. They develop alongside social, political and economic changes and embrace new forms emerging from modern lifestyles. The latter sometimes come into conflict with and challenge the local culture and traditional types of play, which are based on the lives and histories of the indigenous peoples and local communities. A sample of traditional tribal forms of play is analyzed for their health, entertainment and fun aspects. Such play allows children to prepare for life’s realities, in particular for a life of cooperation. By contrast, whilst also providing children with tools and skills for the needs of modern life, new types of play focus more on competition and individualism. This divergence expressed in different types of play widens the generation gap and contributes to alienation. The shift from a collective to individualistic lifestyle thus has an unsettling impact on the community and impacts on the emotional and physical wellbeing of children. We will describe types of play and their role in the holistic development of nomadic children, as well as the impact of modernization and social change, including sedentarization. The article will highlight some consequences of the demise of indigenous play, through observation and analytical comparison of children’s play in three generations. Based on the insights gained, the authors offer recommendations on how to restore traditional play and games through redesigning them to be capable of adaptation to changes in lifestyles.

## 1. Introduction

Recent years have seen the transformation from a traditional society to a modern one for a large part of indigenous nomadic people. Loeffler observed that they are “changing basically the structure of their traditional way of life” alongside “changes in traditional subsistence economy” [1]. The expansion of networking through media has changed many aspects of human lifestyles and habits, especially for parts of the nomadic peoples who have become sedentarized. For both people who have continued a customary lifestyle of mobility or have settled in villages far from urban influences and electricity, traditional systems are still prevalent.

Nationally, while the percentage of migratory indigenous nomadic population has declined from some 50% to around 2%, their absolute number has changed little, vacillating around 1.2–1.5 million. Even large-scale and well-funded national programs of sedentarization [2] have made no dent in this number.

In fact, due to well-established ecological (carrying capacity) and social (governance system and customary laws) reasons, the surplus population of nomads has always migrated out to rural and urban areas, guarding against the stress of overpopulation.

It is to be expected that these societal changes also bring about new forms of play, replacing traditional games with new ones. This understanding is reflected in the United Nations Convention on the Rights of the Child (UNCRC) and has been incorporated in General Comment No. 17 (2013) on Article 31 [3]:

*“Children reproduce, transform, create and transmit culture through their own imaginative play, songs, dance, animation, stories, painting, games, street theatre, puppetry, festivals, and so on. As they gain understanding of the cultural and artistic life around them from adult and peer relationships, they translate and adapt its meaning through their own generational experience. Through engagement with their peers, children create and transmit their own language, games, secret worlds, fantasies and other cultural knowledge.”* 

In this study, we use the term “play” (*baazi* in Luri and Persian) interchangeably [4], both to mean games (structured) and free play (spontaneous, unstructured). Games played in an indigenous and traditional culture are tied to the values of that culture. Whether or not the games that are contrary to the tradition of a society become suitable replacements for traditional games is to be investigated in this paper. Kohgilouyeh and Boyer Ahmad province in Iran was chosen as a focus, because in this province, the acceleration of sedentarization has been more severe than in most other regions of the country. Hence, it provides a microcosm for observing the impact of modernization on children’s behavior in play and consequently in health changes. This province has a deep-rooted nomadic culture, and though many of the nomadic communities have become settled, there are still parts of the tribes that have remained migratory and therefore more traditional.

Among the settled population, the traditional children’s games unique to the Kohgilouyeh and Boyer Ahmad region are gradually disappearing and are being replaced with more generic modern alternatives. We outline the benefits of the old games, many of which could be adapted to suit current lifestyles whilst retaining their spirit of cooperation.

### 1.1. Methodology

Opportunities, as well as challenges, of conducting ethnographic research were highlighted by Friedl, who conducted such research between 1965 and 1994 during eight visits to this tribal region (and spending altogether six years in the village of Deh Koh). She wrote: “…although more than half the population of Iran is under the age of fifteen, few psychologists, educators, and social scientists actually focus on the development, not to speak of the culture, of children…Literature on children is scarce. One can start research anywhere on any topic concerning children, and be a pioneer.” Because children “tend to clam up in the presence of strangers, and they play in hard-to-observe places and rarely with adults, let alone a relative stranger like me” [5], reflecting an indigenous perspective in this paper is invaluable.

This article aims to highlight the importance of the conservation and revival of traditional and local games. We explain the local culture of the area and the role that traditional games play in the development of children’s physical and mental health [6], as well as their practical and social skills. To this end, we compare and contrast the games of three generations of some members of Kohgilouyeh and Boyer Ahmad tribes in the province.

The indicator of the benefits of games for children’s physical and mental health will be the number and kind of social skills children learn in those games. As this piece of work was unfunded and relied on participants’ own, scarce resources, the research tools that might have been employed in a fully-fledged research study were not available to the team. This is why, regarding the first and third generations, the social skills achieved were assessed through a process of reflection, observation and informal discussions. In the second generation, informal discussions were supplemented by the researchers’ own experiences and observations as a second generation nomad. In all generations, participant observation and informal focus group discussions were the primary participatory methods used.

### 1.2. Research History

Many anthropologists have discussed the re-emergence of local games. For example, Ghaffari (2005) [7], an expert in the culture of Kohgilouyeh and Boyer Ahmad, recorded the traditional games of the province. However, up to now, no scientific research has been conducted about the importance of these games and their impact on the health of the children of this province. Our work seeks to provide a first step in this direction, and it is hoped that it will prompt a wider, empirical research study to be conducted in the near future. What distinguishes our emphasis from other approaches is the consideration of social skills in relation to the physical health and mental health aspects of children’s play.

## 2. The Theoretical Basis of the Research

### 2.1. Child Health, Play and Social Skills

Children need particular attention. Social participation by children is very important to their wellbeing and health (Hart, 1997) [8]. UNICEF believes that children’s participation in matters related to them from a very early age helps to protect them in a number of ways: against being involved in deviant activities, getting hurt and being dominated. Their participation contributes to the raising of a generation that feels responsible for and respects their own and others’ rights. UNICEF argues that keeping children at the margins creates a dangerous situation for them. When children are not involved in decision-making processes and in opportunities to act constructively in matters that affect their lives, they do not have the appropriate vital abilities and skills when they grow up (Kamkar, 2003) [9].

This social growth consists of an acquired harmonious collection of social skills and behaviors, which enables individuals to establish good mutual relations with other people, to develop positive responses and to avoid behaviors that have negative consequences. It helps children acquire skills, such as cooperation, responsibility, empathy, self-control and independence, which are important aspects of growth (Gallahue and Ozmun, 1997) [10].

A significant part of a child’s life consists of play and activities discovering the surrounding world. Learning through play is one of the most effective methods of fostering social skills. Play is the child’s world and the focus of its concentration. Through play, children acquire ongoing information about themselves, their bodies and their movements. Many of the basic emotional concepts children develop have their roots in their free and happy world of play (Gallahue and Ozmun, 1997) [10].

Play teaches children to have proper relationships with others: it establishes what “normal” behavior is and promotes cooperation and participation in group activities. The child learns that if s/he has some rights, others should have rights as well. To a large extent, a child’s life in society depends on this relation and activity (Sheikh, 2003) [11].

Experts in developmental theories argue that employing appropriate programs corresponding to the physical and psychological characteristics of human beings, especially during childhood and adolescence, support physical and mental health. Through these processes, individuals are being prepared to live and grow in a social environment (Bellucci, 1980) [12].

James and Prout (1990) [13] believe that children’s play is an important part of their lives. All children are born and grow up in a specific society. Each of these societies consists of individuals who share legal, political and economic structures. An important part of children’s development is to acquire an understanding of these structures. Therefore, when they grow up, they will be able to fit into this social framework. Vygotsky too emphasizes that play has an important part to play in children’s growth, impacting on the development of both cognition and emotional/social development. The role that children play in their games shows their social and cultural position. Through play, children acquire the tools and concepts connected to their culture (Vygotsky, 1976, 1978) [14,15].

Our discussion up to now shows the important role of play in learning social skills and the physical and mental wellbeing of children. In our analysis, the degree of learning social skills through play has been chosen as an index of the physical and mental health of three generations of children in this province.

It must be recognized that in many countries around the world, and for different reasons, there has been a decline in children’s access to play opportunities [16]. Gray concludes that “the decline in play may be the factor that has most directly caused the decline in children’s mental health” and that “restoring children’s free play is not only the best gift we could give our children, it is also an essential gift if we want them to grow up to be psychologically healthy and emotionally competent adults” [17].

### 2.2. Generation

The concept of generation is ambiguous; separating one generation from the next is a difficult task. In this study, there was limited potential for entering the discourse on “generation”. We are addressing three generations of “children” in this province (see below). We acknowledge the fact that the greater the gap between generations, the lesser is the understanding of one generation by the other (Panahi, 2004) [18]. Our division of three generations is based on the kind of game they play or played and the attitudes of children towards these games.

- The first generation includes primarily the children of communities who have been nomadic, as well as those who have settled in villages. Since the 1980s, in some villages closer to cities, television started to make inroads with the spread of electricity. In those areas where a mobile way of life has persisted, this generation of children is still spending their free time playing unique and specific local and traditional games.
- The second generation’s childhood (*ca*. 1980–1990) coincides with the arrival of computer games to the area, but before these became popular and widespread. This generation spent most of their time playing group games and a limited amount of time on computer games. The group games of this generation, such as football, hop-scotch, *haft sang*, dodgeball, *etc.*, were becoming more generic. An important factor in this is the expansion in the means of communication especially through radio and television, which helped disseminate games that are not specific to a certain area or culture.
- The third generation (after 1990) grew up at a time when computer games became widespread and access to the Internet and online games became available. By then, traditional games were largely forgotten by this generation, and the way games are seen has changed in comparison with the two other generations.

Although the games themselves had changed from the first to the second generation, the goals of the games did not change. The games of the second generation were still social and group oriented. However, a marked generational gap can be observed in the differences between the third and first and second generations.

### 2.3. About Kohgilouyeh and Boyer Ahmad Province

#### 2.3.1. Geography

Kohgilouyeh and Boyer Ahmad province is located in southwestern Iran within the Zagros mountain range. It is over 16,000 square km in size and includes eight townships; Boyer Ahmad, Kohgilouyeh, Gachsaran, Dena, Bahmaee, Bashtbavee, Charam and the provincial capital Yasuj. Kohgilouyeh and Boyer Ahmad is known as the “province of four seasons” because of its variable climate and seasonal changes in temperature. This seasonal weather pattern has affected the lifestyle of local people (Ghaffari, 2011) [19] and also the types of play in which children engage.

#### 2.3.2. Culture and Social Position

The provincial dialect is Lori, which is part of the southwestern language group, with the grammar related to Middle-Persian. People of Kohgilouyeh and Boyer Ahmad are nomadic by origin, and the province has hosted Boyer Ahmad, Bahmaee, Tayebi, Bashtbavee, Doshmanziari and Charam tribal confederacies (Ghaffari, 2011) [19]. The lives of nomadic communities are closely tied with nature, and the main source of their livelihood is nomadic pastoralism. Nomads often migrate in large groups of at least “nomadic camps” (*maal*), and the nomadic communities have had specific governance systems; these have broken down or been weakened over the past few decades. This weakening of the tribal systems has resulted in the fragmentation of socio-political frameworks and management arrangements, which used to govern a community as an independent unit. This, alongside numerous other causes, has led to major changes in the lifestyle of the inhabitants of Kohgilouyeh and Boyer Ahmad. One of the most obvious aspects of this upheaval was the large-scale sedentarization of nomadic communities in this region.

Currently, the nomadic population of Kohgilouyeh and Boyer Ahmad can be categorized by their lifestyle as follows:

(i) Migrating camps or “tent-holds” (nomadic households) using traditional means of migration by draft animals, on foot or by car. They practice nomadic pastoralism, but supplement natural rangeland feed with purchased, or sometimes grown, fodder. Living in tents, with challenging environmental conditions, competition with local sedentary populations or occasionally other tribes, and living in harsh mountains meant that individuals in each tribal unit have had to learn to support each other and to have responsibility for each other. It has created a sense of identity in which concepts such as loneliness, alienation or individualism did not exist (Taheri, 2006) [20].

Children from their early years onwards have had to accept some responsibility, according to their age and gender. They learn directly from their daily lives about responsibility and cooperation. For example, very young children are supposed to feed, graze and water chicken, chicks, goat kids and lambs. This is at once a kind of work and a kind of play. Instead of hugging toys, they hug such animals. Therefore, animals play an important role in the lives of these people, and children learn this important fact. Thus, nomadic children, from an early age and through work and play, learn how to enter life in the future when they grow up.

(ii) Sedentarized nomads residing in villages. The change in lifestyle from nomadic to sedentary meant their livelihood was now more reliant upon farming and semi-sedentary animal husbandry.

Although the traditional nomadic culture still exists in parts, as media and other facilities become more accessible in the villages, this culture is starting to diminish.

(iii) Sedentarized nomads residing in cities. These are mainly migrants from villages looking for work. Their employment tends to be less agriculturally based and more in line with that of other city workers, including in the service industries.

## 3. Findings and Discussion

### 3.1. Traditional Local Games (The First Generation Games)

This study took as a sample 33 traditional local games of the Kohgilouyeh and Boyer Ahmad province; of these, nine were chosen to be discussed (see Table 1). From the latter, two are considered in some detail below. The remaining seven, mostly boys’ games, are then listed. Girls would also participate in these games, though we acknowledge that future studies will need to pay special attention to gender and explore girls’ changing play and any impact, positive or negative, on their health.

#### 3.1.1. General Game Rules

Some of the goals and effects of these native games are highlighted in Table 2; many of them demonstrate a spirit of cooperation. Since the rules for designating the “*Salaar*” (game master), electing the game starter and picking team mates are the same for all group games, we begin by describing them in general.

##### Designating the Salaar and Picking Team Mates

At the beginning of each game, two of the oldest players are elected as *Salaar*. The remaining players pair up and decide upon nicknames for themselves. Each pair then puts their arm around one another’s shoulders and introduces themselves to the *Salaar* by giving the two nicknames (without telling which nickname belongs to which player) and saying the word “*sherr*”*.*

One of the *Salaars* replies by saying either “*Andaresh venei gei kol* ” (“It is up to the tail-less cow”), “*Sherr be der*” (torn to pieces) or *“Andar*” (come in). Next, one of the players asks “Do you want the Lion or the Tiger?” and the *Salaar* chooses one of the nicknames. Whichever of the pair it belongs to is the person that joins their team; this will continue until all of the players have been chosen.

In this way, players are chosen anonymously, and none of the children are humiliated by always being the last one to be picked. This increases the self-confidence of children, which is an important factor in their mental wellbeing.

##### Starting the Game

There are several methods to ensure equal chances for either team to start the game: “*Gendi o Qero*”, “*Hali*”, “*Tar o Khoshk*”*:*

*Gendi o Qero:* in this method, the two *Salaars* secretly take on the names “*Gendi*” (meaning lentil in the local dialect) and “*Qero*” (a type of weed growing in lentil fields). They then go to the youngest person and ask “*Gendi bexowse yaa Qero*?” (Should the lentil sleep or the weed?). If the answer is “*Gendi bexowse*” (the lentil should sleep), “*Qero*” is permitted to start the game with his team and *vice versa*.

The reason for using “*Gendi*” and “*Qero*” is that when harvesting lentils, there are *Qero* weeds amongst the lentils that should be pulled out before cooking. This has become a metaphor for picking and sorting. This method both values the decisions of the youngest and provides an equal chance for both parties to start the game.

##### Ending the Game

A team in the winning position can stop the game at any time; to end it in other ways requires an agreement between the teams. This teaches children to play fairly. Therefore, the child learns to respect other people’s rights in a practical manner and through play.

#### 3.1.2. Description of Two Sample Games

##### Qale Tu and Baving Baazi (Family Life and Wedding Game)

In this game, usually played by the children of an extended family or nomadic camp (*maal*), children play out the ordinary life of a family. It usually ends with an imaginary wedding.

Players gather together and build small houses or tents. Each person acts as a family member. To begin with, the children make dolls, farming tools and kitchenware to simulate real life. Young girls usually use the dolls they have made themselves as the bride and groom. The dolls are mainly made of wood and have movable limbs. Girls make them dresses and folk costumes, put colorful cloths in their hands and act out folk dances with them. These dances are an inseparable part of the culture. All of the usual professions within society are played out. At the end of the game, the eldest girl in the group stands in the middle of a circle formed by the other children who sing her questions, which she answers in a “call and response” format song.

##### Kel Kela Bard (Stone Throwing Game)

To play this game, players divide into two teams and stand 20–30 meters apart. Each team places five stones on the ground. The stone closest to the other team is called the “frontier”, representing the commander in war. First, it is agreed who is starting the game. If Team A starts, each team member is allowed to throw one stone at the frontier. Until the frontier is hit, no one is allowed to aim for other stones. As soon as the frontier is hit, aiming for the remaining four stones is allowed. If any of the stones is hit, the player is allowed to throw again.

**Table 1:** Features of some native games.

Name of the Game	Number of Players	Gender of the Players	Necessary Items	Suitable Time to Play	Location
*Qale tu and baving baazi*	2+	Mainly girls, occasionally younger boys	Dolls, farming tools, cooking utensils	No time limit	Near the village or tents
*Kel kela bard*	10+	Boys	Stones	Mild days of the year	Open ground
*Alaxtor*	10+	Boys	None	Days and moonlit nights	Flat open ground
*Jiz*	6+	Boys	A 1.5 to 2 meter-long stick for each player and a spherical stone smaller than a walnut	Days and moonlit nights	Flat, open ground with no pebbles
*Chupishnemaz*	6+	Boys	A walking stick and a cloth for half the players	Days and moonlit nights	Flat open ground
*Chandaani*	2	Girls and boys	28 pebbles or beans	No time limit	Can be played anywhere
*Ḳeri row*	6+	Boys	None	Days and moonlit nights	Open ground
*Qetur*	2+	Mainly girls, occasionally boys	5 small pebbles that fit in the palm of hand of the child	No time limit	Can be played on any flat surface
*Duz*	2	Girls and boys	A flat rock or cardboard sheet, 24 pebbles or beans or sheep/goat droppings	No time limit	Can be played anywhere

**Table 2:** Goals and effects of these native games.

Name	Skill development	The Impact of the Game on Children’s Health
*Qale tu and baving baazi*	Practicing how to build and arrange a house or set up a nomadic tent; also task distribution amongst family members Allows children to practice building and arranging a house or to put up a nomad’s tent, familiarization with household duties, learning how to make food, how to host a guest and teaching the children about familial ties. Teaches the children the costumes and ceremonies, such as traditional weddings, dances, clothing, *etc.*, with the doll making process teaching them how to sew.	Learning life skills and boosting their responsibility
*Kel kela bard*	To practice fighting skills, aiming to throw stones and strengthening their bodies (in nomadic culture, stone, because of its availability, has been commonly used as a weapon).	Strengthening arm muscles, developing concentration and order
*Alaxtor*	Teaching the children how to defend themselves, develop their group work skills, practice keeping balance and building muscular strength.	This game is very active, allowing children to expend their energy
*Jiz*	Develops concentration and reflexes.	Teaches sense of responsibility, increasing concentration and hand-eye coordination
*Chupishnemaz*	Develops the sense of direction.	Increasing concentration and memory
*Chandaani*	Tests and boosts intelligence.	Boosting intelligence and memory and increasing concentration in the players
*Ḳeri row*	Breath-taking and persistence. Develops group work skills.	Strengthens the lungs
*Qetur*	Teaches patience to achieve one’s goals in life.	Teaches discipline and hard work
*Duz*	Develops intelligence and skills for fighting, fight planning and self-defense.	Develops concentration and boosts intelligence and problem solving skills

#### 3.1.3. Classification of the Traditional Games

Based on our examination, we can classify traditional games according to time (some games are only played at specific times), gender, how physical or cerebral they are and if they are accompanied by songs.

Time: *Ti-ti marrow* and *xenaatapak*, in moonlight; *uhala zokch*, in spring and when the earth is damp.

People/tent-holds were living in tents, and the area where children play can be overseen by adults. In villages, people are relatives. This creates a situation in which children can play safely, in moonlight, outdoors. In fact, these night games are only played during moonlight or around the houses. We recognize that nowadays, in some places, children are not allowed outdoors at night. Yet, such play is beneficial for children’s health and well-being such as not being afraid of the dark.

Cerebral games: dooz, chandaani, kus, peshkelagar, kedu.

Physical activity games, mainly played by boys: *alaxtor, jiz gorna doval, band sare leng.*

Games accompanied by singing: *qalah tu va bavig baazi, aqa donba, qelong sarhadi.*

#### 3.1.4. The Characteristics of Traditional Games

The traditional games have developed over time to teach life skills and prepare children for the future. This results in the holistic development of children (including emotional, psychological, physical, social, cognitive, perceptual and moral development).

- These games require no special equipment; thus, they are accessible and affordable for all children regardless of family background and wealth.
- All of the children are equal within the games with the emphasis being on participation.
- The joy and happiness of the players remain whether they win or lose the game.
- Playing in the open air and interacting with the environment, attention to natural elements and dedicating some games to the elements such as rain, develops a sense of responsibility and cooperation, teaches discipline and respect for each other and for nature, prevents selfishness, boosts physical abilities and develops concentration.

Through having identified as an indicator the learning of social skills and their effects on children’s physical and mental health and wellbeing, it can be said that the games of Kohgilouyeh and Boyer Ahmad rank qualitatively high in this indicator.

### 3.2. The Games of the Second Generation

The children of this generation are usually raised in towns and villages. Their childhood coincides with the time that television and radio were becoming widespread and computers began to become available. A gap between parents and children begins to become apparent in this generation, with traditional cultures no longer passed on by parents to the next generation. Nor are teachers and the media aware of the importance of conserving the traditional games. The reasons for this break in the cultural pathway are numerous and beyond the scope of this study.

The children of this generation learn their games from friends and classmates at kindergarten and school; they are greatly influenced by new ideas seen and heard on broadcast media. Therefore, old culturally-specific games start to fade away and are replaced by more general national and globally-available games (these findings are based on observations of the first author of this article, herself a child from the second generation, as well as on interviews with other individuals from the same generation).

Some tent-holds/households do pass down the traditional games to their second generation, but overall, fewer traditional local games are being played in comparison with games played everywhere in Iran. However, because the national games are usually based on traditional culture, in these games at least, there are still some connections to the traditional games. The introduced international and foreign games, on the other hand, have no links with Iranian culture.

Group games, played outdoors, are still popular with this generation. Computer games were not as complex and sophisticated in the past and not as appealing to children as they are today, resulting in less time spent playing them.

Since communities were stronger in the past, there was better supervision over the neighborhood, and the streets were safe places for children to play. Based on the first author’s observations and interviews, the beneficial effects of these games remain evident in the generation.

The positive aspects of collective games in terms of furthering children’s physical and mental health remain also true for this generation.

Since the culturally-rooted traditional games began to fall out of favor with this generation, people became gradually separated from their cultural roots. The games of this generation are not as adept at aiding the development of life skills. For example, Luri singing is being replaced by singing in Persian (the national language, taught exclusively in schools). Therefore, knowledge about the common vocabulary of the local language has been declining. At this time, we therefore witness a kind of social alienation and the beginning of the lack of identity of today’s youth in relation to their local culture and skills. Therefore, it could be said that the games of this generation are contributing much less to improving children’s health than the ones being played by the first generation.

### 3.3. The Games of the Third Generation

Like increasing numbers of children around the world, the children of this generation spend much of their leisure time online, including playing computer games. They rarely play offline group games of any kind; it is only during school breaks that some of these group games can be observed.

Within this generation, there is much less time dedicated to group games in comparison to solitary games. The loss of traditional games in this generation could result from a desire of the young people to adopt what they see as a modern lifestyle with new trends and a new popular culture. This is compounded by the lack of adequate space to play, changes in attitudes towards the aim of games (their spirit now is competitive rather than inclusive and cooperative) and also the increased availability of computer games. Such new forms of play also prepare children for future working life, which increasingly includes work with new forms of technological communication tools. The impact of these rapid changes on both genders of children, and changing parental attitudes, is not yet well understood and requires further exploration.

The scarcity of space for traditional games, different points of view about the goals of games, increased access to computer games and the lack of transmission of one generation’s games to the next are all reasons for a lack of interest in traditional games in this generation. With the fast growth of computer science, computer games have begun to occupy the main part of children’s play. These games have taken the place of collective and other traditional games. On the positive side, these games can increase the imaginative power of children. The child places him/herself in the atmosphere of the game; in their imagination, they consider themselves to be the main champion. In these games, there is a need for speed and fast reaction; therefore, they increase the speed of their hand-eye coordination. One can also teach various educational subjects by these means. Some of these games can be used to fill the leisure time of children. Computer games also have some negative aspects: they can make children agitated; children sit in front of the computer for a long time, and this can have negative consequences for their health (e.g., eyesight, posture). Through reduced physical activity, the child encounters physical damage, including obesity. 

The negative aspect of obesity in Iran has been researched by a growing number of scholars, such as Hemati *et al.*, who wrote: “Unhealthy lifestyle, especially excess time spent on watching TV, may cause obesity in Yasuj [the capital of Kohgilouyeh and Boyer Ahmad] adolescents.” [21]. Others have focused on environmental factors in Iran [22], which are described by Tamjydtash (2011) [23] as follows:

“Increased weight may reduce the level of the child’s activity. This will affect the child’s participation in recreational activities. The increased weight may create problems in terms of acquiring experiences in the general development of the child and will reduce the child’s access to social recreation or play with other children. At the same time, a child’s bodily condition can be an important factor of the child not being socially acceptable.”

In addition to these damages, some children who play online games excessively may lose direct interaction and cooperation with other children. On occasion, their education is also negatively affected. Excessive engagement in such games can damage a child’s emotional relations with parents and other members of their families; this in turn creates and deepens the generational gap and lack of connection between children and parents.

In the authors’ view, modern, mainly computer, games, in terms of social skills, occupy a much lower place on the scale of health indicators compared with traditional games.

## 4. Concluding Observations

### 4.1. Comparing Generations

In the first and second generations, the goal of the games played is defending oneself, one’s tent-hold/household and their belongings, whereas in the third generation, the goal is to demonstrate superiority over playmates. This results in the loss of self-esteem of the loser.

In traditional games, the financial background of a child’s family has no effect on the type of games available. The players have a sense of kinship towards each other, whereas third generation games often require expensive equipment, such as game consoles, which many cannot afford, thus broadening the “digital divide”. This can bring about a sense of selfishness evident in some children’s behavior.

More systematic research is necessary to determine whether violence is increasing in the games of the third generation, in comparison with traditional games, which may have been played in a more or less violent society.

In traditional games, singing is suitable to local culture, while the songs in computer games are suitable to the culture of the society making the game, which occasionally are in contradiction with the local culture. The gender aspects of these changes require further study.

Computer games can sometimes be at odds with cultural values, for instance the way women are depicted can be very different from traditional norms. The potential effects of empowering girls through new forms of play and therefore changing their knowledge levels, status, *etc.*, have yet to be explored. Traditional culture in a given area is an old-established factor, and tensions can arise if changes happen suddenly.

Traditional games prepare children for their future social life by becoming more responsible, being able to communicate well with others and learning traditions from the generations before. Computer games, on the other hand, foster new skills, including communication skills, relevant for today’s educational and employment requirements, but are often based on different values.

Our overall conclusion is that the games of the third generation have different, and arguably reduced, social skills compared with first and second generation games.

### 4.2. Concluding Remarks

Our paper highlights the fact that traditional games drawn from the culture of a people play an important role in teaching social skills to children and in preparing them for their future life. The positive impact of this learning on the health (physical and mental) of nomadic and rural children in Kohgilouyeh and Boyer Ahmad can be observed. Through the passage of time, children have become more interested in modern forms of games. There has been a tendency of children to distance themselves from the traditional play and culture of their people. The effect of these changes on children’s physical and mental wellbeing is vastly under-documented and deserves further study.

### 4.3. Recommendations

To address some of the issues identified, the authors suggest several measures to be considered and implemented:

- The family, a child’s first playmates, should be familiarized with traditional games in order that they can pass these down to the next generation.
- Organizations such as national TV channels, education departments and other relevant bodies have a role to play in transmitting traditional games.
- Traditional games could be included in sport/physical education in schools. Empowering and upskilling teachers is one of the best ways to teach native games.
- Traditional games should be recognized as part of cultural heritage, and the Cultural Heritage Organization (Office of the President of the Republic) should take more actions towards conserving these games.
- Children today live in different circumstances; neighborhoods have changed and there is less space to play. Therefore, updating or adapting traditional games could support their revival and preservation.
- Competitions could be organized that focus on local traditional games and revive those no longer played.
- Since many of the traditional games require large areas of safe, open ground, local parks specifically designed with these games in mind should be created to provide the necessary play space.
